# Overview of statistics teaching within undergraduate programmes in UK and Ireland dental schools

**DOI:** 10.1038/s41415-024-8232-8

**Published:** 2025-02-28

**Authors:** Sam Leary, Neil Cook, Jing Kang

**Affiliations:** 704822062772588332468 Bristol Dental School, University of Bristol, United Kingdom; 553739214768160316698 School of Medicine and Dentistry, University of Central Lancashire, United Kingdom; 800378071997232314487 Faculty of Dentistry, Oral and Craniofacial Sciences, King´s College London, United Kingdom

## Abstract

**Introduction** The United Kingdom (UK) General Dental Council's (GDC's) learning outcomes for undergraduate dental students briefly mention critical appraisal but not statistics. Hence, wide variation in statistics teaching across the dental schools is likely but has not yet been well-documented.

**Methods** A survey was conducted to capture the main features of each of the dental degrees in the 18 UK and Ireland dental schools in terms of statistics-related (standalone or as part of other courses/modules) teaching.

**Results** Representatives from all 18 dental schools completed the survey. There were some similarities, such as most using teaching materials specifically developed for their school, and aiming to teach students to understand/interpret but not generate statistics. However, the number/type of staff delivering the teaching, student contact hours, number of statistical concepts taught, whether statistical theory/formulae/packages were used and inclusion in summative assessments varied extensively. Most reported that this teaching was negatively perceived by the students and many felt that changes were needed.

**Discussion and conclusion** This comprehensive review of undergraduate dental statistics-related provision allows dental schools to compare and contrast their own teaching, which is very timely given the imminent need to implement a new GDC framework. Guidelines to encourage more standardised teaching should be developed to improve the ability of newly qualified dentists to practise evidence-based dentistry.

## Key points

- Wide variation in the provision of statistics-related teaching across undergraduate dental programmes in the UK and Ireland exists.
- Restrictions on time and resources and limited General Dental Council guidance are the main factors driving this variation.
- Guidelines are needed to encourage more standardised statistics-related teaching, which should improve the ability of newly qualified dentists to understand, interpret and critically appraise dental research.

## Introduction

Evidence-based dentistry (EBD) integrates the best available evidence with clinical expertise and patients' needs and preferences to optimise care.^1^ Practising EBD requires the ability to understand and interpret a range of statistical methods used in published research. In addition, statistical errors in published research are common,^2^^,^^3^ so being able to critically appraise the statistical elements of a published paper, as well as other methodological issues, is essential. As highlighted by Sellars,^4^ the majority of dentists are aware of EBD but do not apply it in practice, primarily due to a lack of time and inadequate training. An assessment of statistical knowledge across five health sciences disciplines in the United States of America (USA) identified gaps in knowledge, with staff in dentistry performing worse than those in medicine, nursing, pharmacy and public health.^5^ To our knowledge, this topic has not yet been investigated in the United Kingdom (UK).

The UK's General Dental Council (GDC) Preparing for practice document was published in 2011 and updated in 2015.^6^ This included intended learning outcomes (ILOs) for undergraduate dental students, which focused on EBD, critical appraisal and epidemiology (§1.1.1, §1.1.2 and §1.1.12), but did not specifically mention statistics or data analysis. The new curriculum document - The safe practitioner: a framework of behaviours and outcomes for dental professional education - was published in November 2023,^7^ and will replace Preparing for practice in September 2025. Within the clinical knowledge and skills domain, there are three learning outcomes that mention/imply epidemiology (C1.1, C1.3, C1.27) and one behaviour that mentions an evidence-based approach (C[B]1), and within the self-management domain there is one learning outcome that mentions an evidence-based approach (S2.1) and one that mentions critical appraisal (S2.2), but there is still no mention of statistics. Due to the limited guidance provided, and that some understanding of statistics is necessary to fully critically appraise the evidence, interpretation of these is likely to vary across the UK dental schools.

The provision of statistics teaching in UK undergraduate dental programmes has not been well-documented and most information is now out of date. To our knowledge, the only information available can be obtained from: i) an informal study from 2002 on the teaching of statistics in dental schools based on information received from the 14 dental schools in Britain and Ireland in existence at that time;^8^ ii) an anonymous electronic survey from 2017 focusing on the critical appraisal aspect of EBD completed by 12 of the 16 UK dental schools in existence at that time;^9^ and iii) information from two dental schools (Bristol and Cardiff) included in a summary of the survey of biostatistics teaching in medicine and dentistry in higher education in the UK.^10^

Therefore, a new survey was designed to capture the main features of each of the dental degrees in the 16 UK and two Republic of Ireland dental schools in terms of current statistics-related teaching; this included statistics taught in standalone courses/modules, or as part of other courses/modules, such as research methods, critical appraisal and research projects. The results will provide an opportunity to reflect on current teaching and plan new approaches if needed before full implementation of the new GDC framework in dental curricula.

## Methods

There are currently 14 dental schools in the UK and two in the Republic of Ireland with five-year dental degrees, and two in the UK with four-year dental degrees that are graduate entry only (Table 1).

**Table 1:** UK and Ireland dental schools

Institution	School/faculty	Dental degree
**UK**		
Cardiff University	School of Dentistry	BDS
King's College London	Faculty of Dentistry, Oral and Craniofacial Sciences	BDS
Newcastle University	School of Dental Sciences	BDS
Queen Mary University of London	Barts and The London School of Medicine and Dentistry	BDS
Queen's University Belfast	School of Medicine, Dentistry and Biomedical Sciences	BDS
University of Aberdeen*	Institute of Dentistry	BDS
University of Birmingham	School of Dentistry	BDS
University of Bristol	Bristol Dental School	BDS
University of Central Lancashire*	School of Medicine and Dentistry	BDS
University of Dundee	School of Dentistry	BDS
University of Glasgow	Dental School	BDS
University of Leeds	School of Dentistry	BChD
University of Liverpool	School of Dentistry	BDS
University of Manchester	Faculty of Biology, Medicine and Health	BDS
University of Plymouth	Peninsula Dental School	BDS
University of Sheffield	School of Clinical Dentistry	BDS
**Republic of Ireland**		
University College Cork	Cork Dental School	BDS
Trinity College University of Dublin	School of Dental Science	B DENT Sc
Key: * = Graduate entry only. BDS = Bachelor of Dental Surgery; BChD = Baccalaureus Dentalis Chirurgiae (Bachelor of Dental Surgery); B DENT Sc = Bachelor of Dental Science.		

In 2022, SL (Sam Leary) sought to identify the person most involved in the statistics-related teaching to represent each of the 16 UK dental schools to join a new “dental statistics teachers' group”. This group aims to enhance undergraduate dental education in terms of statistics-related teaching, where ‘statistics-related' refers to any relevant statistics, research methods, critical appraisal or evidence-based-practice teaching. So far, there have been three discussion meetings online and one in person, and the group will become part of the Burwalls Network for Teachers of Statistics in the Health and Life Sciences (https://sites.google.com/view/burwalls/home);^11^ representatives have now been identified from the two Republic of Ireland dental schools and will also be invited to join this network.

After informal discussions regarding variations across the schools, it was decided that a formal survey was required in order to comprehensively capture the current state of statistics-related teaching. A 20-question survey was developed based on these discussions, plus the survey of biostatistics teaching in medicine and dentistry in higher education in the UK summarised by Farnell.^10^ There were a mix of multiple-choice and open-ended questions, covering: the number of students; extent of integration of statistics teaching with other programmes and in the dental curriculum; staff involved in the delivery of the teaching; teaching materials; student contact hours; teaching methods; overall aim; statistical concepts covered; use of theory, formulae and/or statistical packages; assessment methods; recommended additional resources; student perception; restrictions on teaching; and whether it was felt that changes were needed. Ethical approval for this survey was obtained from the University of Bristol (17650), and it was set up as an online survey (https://www.onlinesurveys.ac.uk/).^12^

All 18 representatives from the UK and Ireland dental schools were emailed the survey on 18 April 2024. After reading the survey information, participants were asked to confirm that they agreed to take part in the study. They were asked to indicate their institution would allow a response rate to be assessed, but were told that the institution would be removed before the responses were analysed and would not be included in any dissemination of the results. Up to two reminders to complete the survey were emailed, and the deadline was extended a little to allow three representatives a little more time, closing on 22 May 2024.

All variables were checked for feasible values and completeness. For numerical variables where a range was given, the middle value was selected eg if the number of staff involved in statistics teaching was given as 1-3 then the value 2 was used. Numerical variables were summarised as medians with interquartile ranges (IQRs). Free-text variables, or categorical variables with an option for ‘other' with space for free-text, were coded where possible. Categorical variables were summarised as frequencies and percentages. Free-text variables that could not be categorised were summarised qualitatively. New variables were derived relating to student contact hours and statistical concepts taught. For each year of the curriculum, a binary variable indicating whether or not any statistical components were included was calculated for each school. The total number of student contact hours was calculated for each school by summing the number provided for each year of the curriculum. The participants had been asked to indicate which of a list of 16 statistical concepts were taught in which year of the curriculum. From this, the total number of schools teaching each of the concepts was calculated, along with the total number of concepts taught for each school. All variable derivation and descriptive analysis was undertaken in Stata version 18 (StataCorp).

## Results

### General features of dental schools

The response rate was 100%, with the survey being completed by a representative from all 18 of the dental schools. The median (IQR) approximate number of students per year was 72 (59, 80). The full range was 20-30 for the graduate-entry-only schools, and 35-352 for the remainder. For 27.8% (n = 5) of the schools, dental students were not taught with any other programmes, while in the other (72.2%; n = 13), dental students were taught alongside students from other degrees, including BSc/Diploma in Dental Therapy/Hygiene/Oral Health Sciences, or BSc Bio-Dental Science and Technology/Clinical Dental Technology.

### Where statistics components are taught

Only one school taught statistics as a standalone course, with 61.1% (n = 11) incorporating statistics into research methods/critical appraisal/research projects courses, 11.1% (n = 2) fully integrating statistics into the programme, and 22.2% (n = 4) doing a mix. As there was variation in the extent to which statistics components were integrated into curricula across schools, participants were asked how they would be reporting the statistics components for the remainer of the survey. Most (83.3%; n = 15) reported on the whole research methods/critical appraisal/research projects courses/modules (including problem-based learning sessions), but 16.7% (n = 3) referred to the statistics content only.

### Teaching staff

The median (IQR) approximate number of staff involved in the delivery of the statistics components was 15 (1, 3), with an overall range of 1-7. In half of the schools, there was only one member of staff involved in this teaching.

Table 2 shows the variation across schools in terms of whether at least one staff member involved in the delivery of the statistics components self-identified as having a statistics background, and whether the staff were non-clinical, clinical or a mix. There were 77.8% (n= 14) of schools that had at least one staff member self-identifying as having a statistics background, but only 33.3% (n = 6) had both non-clinical and clinical staff involved in the delivery of the statistics components.

**Table 2:** Type of staff involved in the delivery of the statistics components (total percentages and frequencies are presented)

≥1 self-identified as having statistics background	Non-clinical only	Clinical only	Non-clinical and clinical
Yes	38.9% (n = 7)	11.1% (n = 2)	27.8% (n = 5)
No	0.0% (n = 0)	16.7% (n = 3)	5.6% (n = 1)

### Teaching materials

Although most (88.9%; n = 16) used statistics teaching materials specifically for their school, one used ‘population health' materials, and one linked to general university resources.

### Student contact hours

Table 3 shows the percentage/frequency of schools that did not have any time allocated for the statistics components split by year, and also of those that did have some time, the median (IQR, full range) number of hours in student timetables, split by year and for the whole course. One participant mentioned that students would spend additional time on coursework, and another that students could book individual sessions with a statistician to obtain help with their projects.

**Table 3:** Time allocated for statistics components

Year	% (n) no time	Hours for those with some time:		
		Median	IQR	Full range
1	56.3% (n = 9)*	6.0	3.0, 10.0	1, 27
2	44.4% (n = 8)	5.0	3.4, 7.8	2, 16
3	38.9% (n = 7)	10.0	6.0, 20.0	1, 25
4	50.0% (n = 9)	2.5	1.4, 5.5	1, 6
5	77.8% (n = 14)	3.3	1.4, 4.8	1, 5
Whole course	-	11.8	6.0, 20.6	2, 64
Key: * = Based on n = 16 as no Year 1 for the two graduate-entry-only schools.				

Between 38.9-56.3% of the schools did not include any statistics teaching in Years 1-4, but almost 80% did not include any of this type of teaching in Year 5. Of those schools that did include statistics teaching, the median number of hours ranged from 2.5 in Year 4 and 10.0 in Year 3. Summing across years for each school gave a median of 11.8 hours, with IQR 6.0-20.6 hours for the whole curriculum.

### Teaching methods used

In 22.2% (n = 4) of the schools, only lectures/e-lectures were used for teaching the statistics components. The other schools used lectures/e-lectures in conjunction with either tutorials (44.4%; n = 8), online materials (5.6%; n = 1), or both (27.8%; n = 5).

### Overall aim of the statistics components

The overall aim of the statistics components was for the students to be able to understand and interpret but not generate statistics in 88.9% (n = 16) of the schools. However, two schools (11.1%) aimed for their students to generate as well as understand/interpret statistics.

### Statistical concepts taught

Table 4 shows the number of schools that taught each of the 16 concepts in each year, which ranged from 0-9. The final column of Table 4 shows how many schools taught each concept in any year (one participant did not provide data on which concepts were taught for any of the years for their school). Most commonly taught concepts were summary statistics, p-values, confidence intervals and types of variables, while the least commonly taught concepts were interaction/effect modification, confounding adjustment, assessing agreement and survival analysis. The median (IQR) number of concepts taught across the schools was 10.0 (8.0, 11.5), with an overall range from 5-15 (out of a possible 16 concepts).

**Table 4:** Statistical concepts taught (numbers are n values)

	Year 1	Year 2	Year 3	Year 4	Year 5	Any year (n = 17)
Types of variables	5	4	6	2	1	15
Summary statistics	5	5	6	3	1	17
Sampling	3	6	6	1	1	13
Confidence intervals	4	4	8	5	1	16
P-values	5	4	9	4	2	17
Hypothesis tests	4	3	6	3	1	14
Non-parametric tests	2	0	5	1	1	9
Assessing agreement	1	0	3	0	0	4
Correlation	4	2	4	0	1	10
Regression	3	1	4	1	1	8
Risk ratios	4	4	5	3	1	13
Odds ratios	4	4	5	3	1	13
Confounder adjustment	2	0	0	0	1	3
Interaction/effect modification	1	0	0	0	1	2
Survival analysis	1	1	2	0	1	4
Meta-analysis	1	2	2	5	2	10

There was also an option to list any other statistical concepts taught, which was completed by five of the participants. Additional concepts included sample size/power calculations, sensitivity/specificity, understanding graphs, vital statistics, study design, experimental design, rudimentary probability, the basics of scientific thinking, and scoping/systematic reviews and clinical guidelines.

### Inclusion of statistical theory, formulae and packages

Statistical theory was reported to be included in the teaching for 44.4% (n = 8) of the schools, including central limit theorem/rules of the normal distribution, basics of parametric assumptions, hypothesis testing, descriptive and inferential statistics, and brief description of the main statistical concepts and how/when they are used. There was one comment stating that the teaching was more intuitive than mathematical.

Statistical formulae were reported to be included in the teaching for 33.3% (n = 6) of the schools. These included odds ratios, rate ratios, risk reduction (absolute and relative), number needed to treat, hypothesis tests, correlation, regression and sample size calculations.

Statistics packages were used in 33.3% (n = 6) of the schools. Four Schools used SPSS, one used SPSS and Excel, one used Minitab and one used JASP (n = 1).

### Summative assessments with statistical content

Summative assessments with statistical content were used for 77.8% (n = 14) of the schools. They were reported in Year 1 (n = 2), Year 2 (n = 2), Year 3 (n = 3) and Year 4 (n = 2), with some schools stating more than one year. Summative exams comprised multiple-choice questions (n = 3) and multiple short-answer questions (n = 3). Summative coursework, consisting of tasks such as critical appraisal, project design or reporting data, was reported for Year 2 (n = 2) and Year 5 (n = 1); in one case, the year of the coursework was unclear. Four participants did not provide sufficient detail to allow information to be included in this summary.

### Additional reading suggestions

Additional reading suggestions were provided for 66.7% (n = 12) of the schools. Statistics-focused books with a medical or dental focus (n = 5) such as Essential medical statistics by Kirkwood and Sterne^13^ and Dental statistics made easy by Smeeton^14^ were most commonly recommended. Other textbooks included an epidemiological focus (n = 1), more general statistics (n = 3) and bad science (n = 1). Other sources included a paper series on EBD, critical appraisal tools/guidance, online resources and YouTube videos; although, little detail was provided on what these comprised.

### Student perception of the statistics components (n = 16)

Participants reported that students had positive perceptions in four of the schools, and only one reported active dislike. Three reported varied opinions but tended to lean more towards negative perceptions. The most commonly reported issues were students not seeing the relevance of the subject matter (n = 6), or that they simply were not interested (n = 2). There were comments that enjoyment and engagement may be improved by tailoring the content to them as dental professionals (n = 1) and focusing more on interpretation of data than carrying out statistical tests (n = 1). Contributors to negative viewpoints were the content being perceived as difficult (n = 5) and student apprehension about unfamiliar content (n = 1).

### Restrictions on what/how the statistics components are taught

No restrictions were reported by participants in 33.3% (n = 6) of the schools. In the other schools, the main restriction mentioned was time (n = 8). The content taught was reported to be chosen or guided by others by four of the participants, and two mentioned a lack of specialist teaching staff. Other issues highlighted were inappropriate rooms being allocated (n = 1) and students being overworked and focusing on clinical matters (n = 1).

### Suggested changes for teaching the statistics components

Only one participant did not feel that any changes were necessary, owing to a supportive school that values the material being taught. Two felt that they were doing the best they could with the time allocated and another was unsure if any changes were required. Participants from the other schools suggested a range of improvements, including: more practical application (n = 4); re-distribution of the teaching content into either starting earlier or using an approach which spreads the teaching of skills across the entire course (n = 4); greater integration of statistics teaching (n = 3); increasing dental relevance, potentially with greater clinician input (n = 3); and allowance for more depth and/or time (n = 2). Two schools additionally suggested making the content either entirely optional for those interested or including optional additional depth. One school suggested including more assessment.

### Other comments

Additional comments regarding teaching the statistical components were provided by six of the participants. Additional points not made previously included the difficulty in pitching the level of the content to a mixed group of students (eg for graduate-entry-only schools, or if teaching is shared between undergraduate programmes), and problems with staffing due to this content being perceived as low priority.

## Discussion

This survey capturing the main features of undergraduate dental degrees in terms of statistics-related teaching was completed by a representative from all 18 of the UK and Ireland dental schools. Wide variation across schools was seen for the number/type of staff delivering the teaching, student contact hours, number of statistical concepts taught, whether statistical theory/formulae/packages were used and type/timing of summative assessments, including statistical content. There was some variation in terms of whether dental students were taught with students from other programmes, the extent of integration of the statistical teaching into the rest of the programme, teaching methods used and whether any additional reading suggestions were provided. Consistency was only reported for using teaching materials specifically developed for their school, and aiming to teach students to understand/interpret but not generate statistics. Most reported that this teaching was negatively perceived by the students, and many felt that changes were needed, primarily increasing the time and resources available for delivery of this teaching.

It is not surprising that many aspects of the statistics-related teaching in dental undergraduate curricula vary widely due to the limited guidance available from the GDC. The informal statistics teaching study undertaken by Smeeton in 2002^8^ also found that there was variation in which year of the programme the statistics teaching took place, student contact hours, teaching methods used, whether statistical packages were used, assessment methods used and whether additional reading suggestions were provided. The 2002 survey reported that dental departments only took responsibility for the statistics teaching in 36% of the schools. The dental school representatives that completed the current survey were almost all (94%) based in dental schools and, as relevant examples are essential for clinical students' understanding of statistics,^15^ this is a welcome improvement.

In 2017, Hong and Plugge^9^ also found very varied approaches for all the critical appraisal aspects of EBD that were surveyed, namely which year of the programme the teaching took place, student contact hours, teaching methods and assessment methods used. According to Smeeton,^8^ very little is known about statistics teaching outside the UK and Ireland, and to our knowledge, this has not changed in more recent years. Some information is available regarding evidence-based practice teaching, such as the 2024 systematic review based on 12 studies, half of which were based in the USA,^16^ but there is no specific mention of statistics.

It is possible to include statistics-related teaching into a dental undergraduate curriculum successfully, both in terms of student engagement and also demonstration of skills learnt.^17^^,^^18^ However, this requires leadership from someone with a statistics background who has a substantial amount of time to invest in designing the teaching, other staff (ideally at least one clinician to promote the clinical relevance) to assist with delivery of the teaching, e-learning support, and crucially, an appreciation of the importance of this teaching from senior dental school staff to ensure adequate time can be allocated in the curriculum. One of the survey participants commented that the difficulty of teaching statistics can only be appreciated by those that teach it themselves, which may partly explain the lack of time allocated in many schools.

Developing guidelines to encourage more standardised statistics-related teaching should lead to an improvement in the ability of newly qualified dentists to practise EBD in the long-term. The Association for Dental Education in Europe is currently running an open consultation for their new research domain for the Graduating European Dentist;^19^ their intended learning outcomes do not specifically mention statistics but do cover critical appraisal and EBD in detail, so they could be a helpful starting point. Statistical guidelines for adequately reporting findings from oral health research have recently been published as part of a collaborative effort between some of the key journals in the field;^20^^,^^21^ these guidelines could be used as a basis for discussion regarding which statistically related concepts should be included in undergraduate teaching. Although relating to life sciences in general rather than dentistry specifically, a relevant discussion regarding the depth of statistics that should be taught could also be considered.^22^ Ideally, a Delphi study,^23^ a technique that has previously been used in dental education (eg Khalaf *et al.*),^24^ should be undertaken to allow consensus to be reached on the specific translation of the GDC framework into undergraduate dental curricula in terms of statistics-related teaching. It will, however, be essential to bear in mind that the overall number of learning outcomes, and in some cases, the depth of knowledge required, has increased in the new framework, yet the length of undergraduate dental degrees will remain the same.

There were some limitations of the current study:

- Variation in the extent to which the statistics components were integrated into curricula will have affected some of the results, such as the total number of student contact hours. However, participants were asked to indicate whether they were referring to the statistics-only content, or the whole research methods/critical appraisal/research projects courses/modules, which included some statistics content, so this aided interpretation of their responses
- There may have been some ambiguity with interpretation for some of the questions, but allowing free-text was intentional eg participants could self-identify as having a statistics background rather than having to indicate whether or not they held a statistics qualification
- It was not possible to capture every feature of statistics-related teaching, for example, staff turnover, so these findings might not be fully reflective of this teaching in the UK and Ireland.

Despite the limitations, to our knowledge, this is the first survey to capture the main features of undergraduate dental degrees in terms of statistics-related teaching, which achieved 100% participation rate from the dental schools in the UK and Ireland, with very little missing data. These results are therefore the best reflection available on statistics-related teaching at the current time, and can also offer reference for countries outside the UK and Ireland, should they wish to undertake a similar survey.

## Conclusion

In conclusion, this comprehensive review of undergraduate dental statistics-related provision allows dental schools to compare and contrast their own teaching, which is very timely given the imminent need to implement the new GDC framework which has necessitated curriculum review. The survey findings may provide reassurance for those who are satisfied with their current teaching but also evidence of what is possible with enough resources for those who are struggling to achieve more comprehensive statistics-related teaching in their school. Improving this teaching, through development of a new set of detailed guidelines based on consensus achieved via a Delphi approach should ultimately enhance patient care. Further work should include review of statistics-related teaching for other undergraduate and also postgraduate programmes in dental schools in the UK and Ireland.

## Data Availability

We do not have ethical approval to share the data on which this publication was based.
